# Experiences of people seen in an acute hospital setting by a liaison mental health service: responses from an online survey

**DOI:** 10.1186/s12913-021-06974-4

**Published:** 2021-10-05

**Authors:** Elspeth Guthrie, Daniel Romeu, Carolyn Czoski-Murray, Samuel Relton, Andrew Walker, Peter Trigwell, Jenny Hewison, Robert West, Matt Fossey, Claire Hulme, Allan House

**Affiliations:** 1 Leeds Institute of Health Sciences, University of Leeds, Leeds, UK; 2 Leeds and York Partnership NHS Foundation Trust, Leeds, UK; 3 Clinical Research Network National Coordinating Centre, National Institute of Health Research Clinical Research Network, Leeds, UK; 4 National Inpatient Centre for Psychological Medicine, Leeds and York Partnership NHS Foundation Trust, Leeds, UK; 5 Veterans and Families Institute for Military Research, Faculty of Health, Social Care and Education, Anglia Ruskin University, Chelmsford, UK; 6 College of Medicine and Health, University of Exeter, Exeter, UK

**Keywords:** Liaison Psychiatry, Mental Health Liaison Services, Qualitative, Experiences of care

## Abstract

**Background:**

In recent years the UK has expanded the provision of liaison mental health services (LMHS). Little work has been undertaken to explore first-hand experiences of them.

**Aims:**

The aim of this study was to gain insights into the experiences of users of LMHS in both emergency departments and acute inpatient wards in the UK.

**Methods:**

This cross-sectional internet survey was initially advertised from May-July 2017 using the social media platform Facebook. Due to a paucity of male respondents, it was re-run from November 2017-February 2018, specifically targeting male respondents. The survey featured a structured questionnaire divided into three categories: the profile of the respondent, perceived professionalism of LMHS and overall opinion of the service.

**Analysis:**

Responses to the structured questionnaire were analysed using descriptive statistics and latent class analysis. Free-text responses were transcribed verbatim and interpreted using thematic analysis.

**Results:**

184 people responded to the survey. 147 were service users and 37 were partners, friends or family members of service users. Only 31% of service users and 27% of close others found their overall contact helpful. Latent class analysis identified three clusters − 46% of service users generally disliked their contact, 36% had an overall positive experience, and 18% did not answer most questions about helpfulness or usefulness. Features most frequently identified as important were the provision of a 24/7 service, assessment by a variety of healthcare professionals and national standardisation of services. Respondents indicated that the least important feature was the provision of a separate service for older people. They desired faster assessments following referral from the parent team, clearer communication about next steps and greater knowledge of local services and third sector organisations.

**Conclusions:**

This survey identified mixed responses, but overall experiences were more negative than indicated in the limited previous research. The evaluation and adaptation of LMHS along the lines suggested in our survey should be prioritised to enhance their inherent therapeutic value and to improve engagement with treatment and future psychiatric care.

**Supplementary Information:**

The online version contains supplementary material available at 10.1186/s12913-021-06974-4.

## Introduction

Liaison mental health services (LMHS) provide mental healthcare to people in the acute hospital setting - in emergency departments, general ward settings, and less often in specialist outpatient clinics. In the UK, there has been a gradual expansion of LMHS over recent years, such that now every acute hospital in England with an emergency department has a liaison service [1].

A recent review of experiences of acute hospital care by people with mental health problems identified sixteen previous studies which focused on the emergency department setting [2]. The review divided experience according to structure (setting and staff), process and outcome (satisfaction). Most of the studies reported negative experiences of the emergency department, describing it as overstimulating and lacking in comfort and privacy [3, 4]. Participants in the studies also described it as feeling unsafe [5] and lacking basic hygienic amenities [6]. Staff were described in largely negative terms, such as uninterested or intimidating [3], and lacking expertise concerning patients’ diagnosis [4, 5]. Although Harris and colleagues examined experiences of emergency department staff and not liaison staff [3], these experiences remain important as an individual seeking mental health support in the emergency department will likely have contact with both emergency and LMHS teams, both of which may influence experience and subsequent health-seeking behaviour. In terms of process, patients generally felt dissatisfied with their care and lacked information about aftercare, such as self-help groups or telephone helplines.

Another review of service users’ experiences in the emergency department identified nine studies [7]. Services users described stressful experiences with long waiting times [2]. Their experiences of liaison staff were mixed: some described staff as judgmental while others described staff as caring and placing people at ease [8]. The small number of studies that included or focused on service users’ experiences of LMHS generally reported positive interactions with staff, including being listened to, reassured and consoled [4, 8]. Two recent studies from Australia have also reported high levels of satisfaction from users of an emergency department-based mental health liaison nurse service [9, 10].

In the UK there has been little work to understand service users’ experiences of LMHS. In a few studies questionnaires have been used to evaluate service user satisfaction with LMHS, which have generally been positive [11–13]. The only study to our knowledge in which rigorous qualitative methods were used to assess service user experience is that of Eales and colleagues who carried out semi-structured interviews with seventeen patients who had used LMHS [14]. The main author then conducted more thorough secondary analysis of the data in 2013 [4]. Service users described long waits before being able to access LMHS, during which people described a sense of distress, fear and hopelessness. When people saw a liaison practitioner their experiences varied. A therapeutic experience was associated with the service user being helped to understand and make sense of their psychological crisis, which included: a sense of connection with the mental health practitioner; being taken through a process at a pace that was comfortable for the service user; understanding the essence of the service user; accounting for and exploring the person’s history in the context of the current problem including physical as well as mental health; conferring with others to check what is normal for the service user; and involving the service user in decision making.

Studies from Australia have generally reported positive experiences of LMHS. Wand and colleagues interviewed twenty-three participants who had experience of a large nurse-led liaison service in Sydney [8]. Participants reported numerous therapeutic benefits from the service including feeling listened to, understood and welcome, a focus on solutions not problems, a sense of hope and optimism deriving from the experience and timely and easy access to the service. Summers and Happell described high levels of satisfaction with availability of LMHS staff and their ability to provide support and care at a hospital in Melbourne [15]. Callaghan and colleagues surveyed users of LMHS in England in 2002 and received seventy-one responses, representing 16 % of those surveyed [16]. In general responses were favourable, as were those by Wand and colleagues in Australia [17], although the poor response rate limits the generalisability of the findings.

All studies mentioned above describing service users’ attitudes to LMHS have been single site studies of LMHS sited in emergency departments. A recent exception is Wand and colleagues’ recent work evaluating emergency-department nurse-led liaison services in rural sites [18]. It is possible that the desire to understand service user experience is associated with high quality LMHS and also that responders to such studies are more likely to have had a favourable experience of care than non-responders.

The aim of the present study was to gain insights into the full range of service users’ experiences of liaison mental health services both in the emergency and general hospital setting using the internet rather than approaching service users via existing services. This method was selected as an inexpensive means of accessing large numbers of potential respondents who had used different services across England. It is not possible to determine the representativeness of study populations recruited via the internet, as the size of the denominator is unknown, so we regarded findings from the survey as indicating the nature of service user experience rather than as indicators of the frequency of such experiences.

## Methods

This work formed part of the first phase of a programme funded through the NIHR Health Services and Delivery Research scheme to evaluate the cost-effectiveness and efficiency of different configurations of liaison psychiatry services in England (LP-MAESTRO) (http://www.nets.nihr.ac.uk/projects/hsdr/135808).

### Definitions

In our definition of liaison mental health services we have included both ward-focused services, where professionals provide care for mental conditions in those with pre-existing physical health issues in the general hospital setting, and the acute liaison psychiatry service, where professionals deliver one-off assessments for those presenting to the emergency department with an acute mental health problem or following an act of self-harm and may also provide self-harm assessments in the inpatient ward setting. The professionals involved are varied and typically include nurses, psychiatrists and other allied health professionals, but not usually clinical psychologists.

### Design

The present study is a cross-sectional internet survey exploring the experiences of users of LMHS via the social media platform Facebook. In 2016, Facebook was the most used social media platform globally, with over two billion active accounts by 2017 [19], and it was selected to maximise the likelihood of obtaining rich and varied data. In addition, Facebook’s established survey advertising system with filter and keyword selection options contributed to the decision, alongside the research team’s familiarity with the site.

### Ethics approval and consent to participate

Ethical approval was received from the North of Scotland Ethics Research Service (REC reference: 15/NS/0025). Completion and submission of the questionnaire was taken to imply that consent for the use of the questionnaire data has been granted for the purposes stated in the information about the research and its aims.

### Data collection

Data were collected using an online survey questionnaire. This was disseminated via Facebook advertising on two occasions and shown in Appendix 1. It was initially run from May – July 2017. Due to a paucity of responses from male service users on the first round, the survey was re-run with a focus on this demographic using Facebook filters from November 2017 – February 2018. Questions were divided into three main categories: the profile of the respondent, perceived professionalism of LMHS, and overall opinion on the service. Space was available for free-text comments in each section.

### Data analysis

Principal analysis of the survey data was undertaken with R statistical software V.3.2.2 (R Core Team 2016). Quantitative, descriptive data of numbers and percentages are reported for the Facebook survey.

A latent class model was fitted to cluster responses of service users into interpretable groups using the poLCA package. The number of clusters was determined by minimising the Bayesian information criterion (BIC) which favours minimisation of complexity.

Free-text comments were transcribed verbatim and analysed using thematic analysis [20]. Familiarisation with the data was followed by identification of a thematic framework, indexing, charting, mapping and interpretation. This work was initially carried out by A.H. and verified by E.G. and D.R.. Responses were synthesised into themes: logistics of LMHS contact; attitudes and behaviour of staff; communication with liaison staff; in hospital intervention; aftercare; and characteristics of a desirable service. Where appropriate, we have followed the Consolidating Criteria for Reporting Qualitative Research (COREQ) guidelines [21].

## Results

In total there were 184 respondents to the Facebook survey, of which 147 (79.9%) were service users and 37 (20.1%) were partners, relatives or friends of a service user.

Measures of audience interactions with the Facebook advertisement are shown in Table 1. The survey completion rate for those who clicked on the advertisement was 17% (184 respondents out of 1116 clicks), split by gender as 19% for females (155/822) and 8% for males (23/277), excluding six participants who did not disclose their gender. Data on gender were missing for seventeen clicks. Of those who clicked on the advert eighty-four came from Facebook Messenger whilst thirty-four accounts chose to share our advertisement with their friend network, further expanding the reach of the survey.

**Table 1:** Survey interaction measured by clicks, views, and number of on-screen appearances

	Round 1	Round 2 – Male Only	Total
**Clicks**	885	231	1116
**Views**	44,281	16,571	60,852
**On-screen**	88,582	25,551	114,133

### Profile of survey respondents

The profile of all survey respondents is summarised in Table 2 and details the distribution of age, recent contact with LMHS, reason for attendance, LMHS staff seen, and site of contact. Of the 147 survey respondents who were service users, the majority were aged 34 years or less (85, 58%) and most identified as female (126, 86%). 58 (40%) service users had contact with LMHS in the six months prior to their current presentation and time since last LMHS contact ranged from 4 h to 13 years. 82 (56%) service users had attended with consequences of self-harm, 42 (29%) presented with a mental health problem, and more than half were worried about their mental health at the time of referral to LMHS (80, 54%). The majority (77, 52%) were seen by a mental health nurse and almost one third (46, 31%) were seen by a psychiatrist. Approximately half of contacts were in the emergency department (71, 48%) and slightly more were on a ward setting (73, 50%). Some respondents were assessed by LMHS in multiple sites. Over three-quarters (112, 76%) received a biopsychosocial assessment.

**Table 2:** Baseline characteristics of survey respondents

Variable	Categories	Service users (N = 147)		Friends, family and partners (N = 37)	
		**Number Missing (%)**	**Number (%)**	**Number Missing (%)**	**Number (%)**
**Age**		1 (0.7)		0 (0)	
	18–24 years		54 (36.7)		6 (16.2)
	25–34 years		31 (21.0)		1 (2.7)
	35–44 years		27 (18.4)		12 (32.4)
	45–54 years		24 (16.3)		12 (32.4)
	55–64 years		9 (6.1)		6 (16.2)
	65 + years		1 (0.7)		0 (0)
**Gender identified with**		0 (0)		0 (0)	
	Female		126 (85.7)		29 (78.3)
	Male		16 (10.9)		7 (18.9)
	Would rather not say		5 (3.4)		1 (2.1)
**Contact with LMHS in previous 6 months**		29 (19.7)		11 (29.7)	
	Yes		58 (39.5)		16 (43.2)
	No		60 (40.8)		10 (27.0)
**Reason for hospital attendance**		0 (0)		0 (0)	
	Mental health problem		42 (28.6)		21 (56.8)
	Physical health problem		22 (15.0)		7 (18.9)
	Consequence of self-harm		82 (55.8)		9 (24.3)
	Pre-operative assessment		1 (0.7)		0 (0)
**Worried about mental health at time of referral**		0 (0)		1 (2.7)	
	Yes		80 (54.4)		21 (56.8)
	No		53 (36.1)		7 (18.9)
	Not sure		8 (5.4)		3 (8.1)
	Not applicable		6 (4.1)		5 (13.5)
**LMHS staff seen**^**1**^		0 (0)		0 (0)	
	Allied Health Professional		3 (2.0 %)		1 (2.7)
	Mental Health Nurse		77 (52.4)		13 (35.1)
	Not applicable		13 (8.8)		4 (10.8)
	Not sure		22 (15.0)		9 (24.3)
	Other		11 (7.5)		3 (8.1)
	Psychiatrist		46 (31.3)		8 (21.6)
	Psychologist or Psychotherapist		15 (10.2)		5 (13.5)
**Site of contact with LMHS**^**2**^		0 (0)		0 (0)	
	Emergency Department		71 (48.2)		12 (32.4)
	Ward		73 (49.7)		17 (46.0)
	Pre-operative clinic		9 (6.1)		3 (8.1)
	Not sure		1 (0.7)		1 (2.7)
	Not applicable, or answered ‘no’ to all sites		0 (0)		16 (43.2)
**Had biopsychosocial assessment**		1 (0.7)		2 (5.4)	
	Yes		112 (76.2)		27 (73.0)
	No		22 (15.0)		4 (10.8)
	Not sure		12 (8.2)		2 (5.4)
	Not Applicable		0 (0)		2 (5.4)

^1^The percentage for LMHS staff seen do not add up to 100 because some participants selected multiple staff (e.g., nurse and psychiatrist). ^2^The percentage of contact with sites do not add up to 100 % as some participants selected ‘yes’ to multiple sites

The age of survey respondents who were partners, family members of friends or service users (N = 37) ranged from 18 to 64, and 24 (65%) were aged 35–54. The majority (29, 78%) were female. 21 (57%) reported that the service user had presented with a mental health problem and 9 (24%) with consequences of self-harm. Most (21, 57%) were worried about the service user’s mental health at the time. LMHS staff seen included mental health nurses (13, 35%), psychiatrists (8, 22%), psychologists or psychotherapists (5, 14%) and other allied health professionals (1, 3%). 12 (32%) reported that the service user was seen in the emergency department and 17 (46%) were seen on the ward. Nearly three quarters (27, 73%) reported the service user to have received a biopsychosocial assessment.

### Perceived professionalism of LMHS

Data regarding the survey respondents’ perceived professionalism of LMHS staff are presented in Table 3. Of the 147 service users, 91 (62%) reported that LMHS staff explained why they had been called and only 84 (57%) reported that LMHS staff introduced themselves. The majority (91, 62%) felt that an assessment from the liaison team was needed before their discharge from hospital. For just over one third of service user respondents (52, 35%) did the LMHS assessment come at a good time. The time reported between arrival at hospital and contact with the LMHS team ranged from “immediately” to “several weeks”. In two thirds of cases (97, 66%) the assessment was completed in a private space, but only 23 (16%) were offered advocacy or support outside of the wider medical team. Fewer than half of service user respondents reported receiving help going forward – 40 (27%) were signposted to other services and 58 (40%) were offered outpatient assessment, although (3%) reported to have self-discharged.

**Table 3:** Survey respondents’ views on the professionalism of LMHS

Variable	Responses	Service users (*N* = 147)		Friends, family and partners (*N* = 37)	
		**Number missing (%)**	**Number (%)**	**Number missing (%)**	**Number missing (%)**
**LMHS staff explained why they had been called**		1 (0.7)		1 (2.7)	
	Yes		91 (61.9)		22 (59.5)
	No		21 (14.3)		4 (10.8)
	Not sure		16 (10.9)		5 (13.5)
	Not applicable		18 (12.2)		5 (13.5)
**Thought needed LMHS assessment before would be discharged**		1 (0.7)		0 (0)	
	Yes		91 (61.9)		20 (54.1)
	No		27 (18.4)		6 (16.2)
	Not sure		12 (8.2)		4 (10.8)
	Not applicable		16 (10.9)		7 (18.9)
**LMHS staff introduced themselves**		2 (1.4)		0 (0)	
	Yes		84 (57.1)		20 (54.1)
	No		29 (19.7)		6 (16.2)
	Not sure		18 (12.2)		6 (16.2)
	Not applicable		14 (9.5)		5 (13.5)
**Assessment came at a good time**		21 (14.3)		5 (13.5)	
	Yes		52 (35.4)		8 (21.6)
	No		48 (32.7)		9 (24.3)
	Not sure		23 (15.6)		11 (29.7)
	Not applicable		3 (2.0)		4 (10.8)
**Assessment done in a private space**		21 (14.3)		3 (8.1)	
	Yes		97 (66.0)		22 (59.5)
	No		22 (15.0)		7 (18.9)
	Not sure		4 (2.7)		2 (5.4)
	Not applicable		3 (2.0)		3 (8.1)
**Offered advocacy during contact**		0 (0)		0 (0)	
	Yes		23 (15.6)		13 (35.1)
	No		100 (68.0)		18 (48.6)
	Not sure		11 (7.5)		2 (5.4)
	Not applicable		13 (8.8)5		4 (10.8)
**Signposted to other services**		1 (0.7)		1 (2.7)	
	Yes		40 (27.2)		12 (32.4)
	No		85 (57.8)		18 (48.6)
	Not sure		14 (9.5)		2 (5.4)
	Not applicable		7 (4.8)		4 (10.8)
**Offered outpatient follow-up**		0 (0)		0 (0)	
	Yes		58 (39.5)		15 (40.5)
	No		65 (44.2)		14 (37.8)
	Not sure		1 (0.7)		1 (2.7)
	Not applicable		8 (55.4)		4 (10.8)
	Left without seeing anyone		10 (6.8)		1 (2.7)
	Self-discharged		5 (3.4)		2 (5.4)

### Overall opinions of LMHS

Over a quarter of service users (40, 27%) wanted to see a member from LMHS but were not given the opportunity. 23 service users (16%) thought that their physical health treatment was dependent on an assessment from the liaison team. Only a small number of service user respondents reported that they felt understood (35, 24%) and comfortable (43, 29%) during their assessment. Just over one fifth of service users (31, 21%) and friends, family and partners (8, 22%) found the initial assessment helpful and nearly one third of service users (46, 31%) and close others (10, 27%) found overall contact with LMHS helpful. Table 4 summarises further data detailing all survey respondent’s overall opinions of LMHS.

**Table 4:** Survey respondents’ overall opinions of LMHS

Variable	Responses	Service users (*N* = 147)		Friends, family and partners (*N* = 37)	
		**Number missing (%)**	**Number (%)**	**Number missing (%)**	**Number missing (%)**
**Wanted to meet LMHS staff but did not get the chance**		2 (1.4)		2 (5.4)	
	Yes		40 (27.2)		20 (54.1)
	No		6 (4.1)		2 (5.4)
	Not sure		9 (6.1)		1 (2.7)
	Not applicable		90 (61.2)		12 (32.4)
**Thought their physical treatment was dependent upon them seeing LMHS services**		23 (15.6)		4 (10.8)	
	Yes		23 (15.6)		4 (10.8)
	No		58 (39.5)		14 (37.8)
	Not sure		15 (10.2)		6 (16.2)
	Not applicable		28 (19.0)		9 (24.3)
**Felt understood**		20 (13.6)		4 (10.8)	
	Yes		35 (23.8)		9 (24.3)
	No		69 (46.9)		12 (32.4)
	Not sure		21 (14.3)		8 (21.6)
	Not applicable		2 (1.4)		4 (10.8)
**Felt comfortable**		22 (15.0)		4 (10.8)	
	Yes		43 (29.3)		14 (37.8)
	No		68 (46.3)		13 (35.1)
	Not sure		10 (6.8)		3 (8.1)
	Not applicable		4 (2.7)		3 (8.1)
**Found initial LMHS assessment helpful**		16 (10.9)		4 (10.8)	
	Yes		31 (21.1)		8 (21.6)
	No		75 (51.0)		14 (37.8)
	Not sure		18 (12.2)		7 (18.9)
	Not applicable		7 (4.8)		4 (10.8)
**Found overall LMHS contact helpful**		1 (0.7)		0 (0)	
	Yes		46 (31.3)		10 (27.0)
	No		70 (47.6)		15 (40.5)
	Not sure		17 (11.6)		6 (16.2)
	Not applicable		13 (8.8)		6 (16.2)

All survey respondents, including service users and close others (N = 184) were asked to choose the most important feature of LMHS from a list of set responses. The features most frequently identified as important were: offering a 24/7 service (72, 39%); having a variety of different healthcare professionals (41, 22%); standardising services nationally (18, 10%); and offering a referral to community services (17, 9%). The least important features reported were: having a separate service for older patients (91, 50%); standardising services nationally (26, 14%); having target response times (18, 12%); and LMHS staff sharing more information with the physical health team (17, 9%)

### Clusters

The survey responses of service users (N = 147) were clustered using latent class analysis. Given the number of respondents and questions we were able to fit models with two, three and four classes. Three latent classes provided the best fit for the data (via the AIC, BIC, log-likelihood, and Chi-squared metrics) and the classes are clearly separated in terms of their opinions.

Class 1 contained 68 (46%) of service users who generally disliked their experience with LMHS. They found the service unhelpful and did not feel understood or comfortable. Their recommendations for developing LMHS were to offer a 24/7 service and to have a variety of different types of staff available. They were least concerned about a separate liaison service for older people.

Class 2 contained 53 (36%) service users who found their interaction with the service helpful and felt understood and comfortable during their experiences. They also indicated that the provision of a 24/7 service and a mix of staff were important to them and did not feel that a separate service for older people was important to them.

Class 3 contained 26 (18%) respondents who did not answer most of the questions about the usefulness and helpfulness of LMHS, thus we were unable to ascertain their opinions with any degree of confidence.

### Free text responses

#### Experience of the initial contact

Several respondents commented on the long wait to see somebody, even when referred from the emergency department, for an interview that was then short, rushed and was not always conducted in conditions that guaranteed privacy.

*“It was a 10-minute chat which followed a long wait”*

On the other hand, positive comments mentioned being given the time and space to talk freely once contact was made. It was helpful that staff returned later when they found a respondent in no state to talk freely at initial contact.

*“They came roughly when they said they would and they contacted the correct people afterwards”.*

*“They gave a full assessment and took relevant action and showed care while I was waiting for a hospital transfer”.*

#### Attitude and behaviour of staff

There are many comments on this theme. Positive comments from respondents described feeling listened to, treated with kindness and respect and met by somebody who seemed interested and understanding.

*“The doctor listened, was kind and treated me like a “normal” person rather than using the patronising tone often adopted in the CMHT”*.

Negative comments, however, described the liaison clinician as dismissive, patronising, disinterested, lacking in respect and going through the motions. Sometimes the attitude was more actively unpleasant and critical.

*“They could have listened, cared, acted interested, believed what I was saying instead of literally dismissing everything”.*

*“The psychiatrist told me I should be grateful I was a student at [university name] and told me to consider myself lucky as many people would love that opportunity”.*

#### Communication with LMHS staff

Negative comments were a continuation of the theme about poor attitudes. Respondents reported being asked the same questions repeatedly in what felt like an over-structured interview that placed excessive attention on the assessment of mental capacity and risk.

*“People skills were hugely lacking. No attempt made to help me calm down and very quick to make me someone else’s problem”.*

*“I felt trapped and they used sectioning as a threat to keep me in but didn’t offer any help”.*

By comparison, positive features of communication involved a more interactive and exploratory style, a willingness to meet family and to communicate with the main clinical team.

*“Very polite and interested, received a letter with the details of my assessment”.*

### In hospital intervention

For several respondents, the contact was noted as a helpful opportunity to talk about their problems.

*“They listened to me, they don’t judge me and I got the help I needed”.*

*“Helped me see it was an illness and not that I wasn’t strong enough”.*

Respondents valued the opportunity to review options, even about something as simple as medication change, which several respondents mentioned as helpful. Discharge was supported by referral to community services such as crisis care or giving contact details for statutory or third sector groups.

The commonest complaint was that assessment led to no action at all, not even information about what might be available.

*“Nothing in my eyes, I get more help talking to the voices”.*

### Aftercare

The main issue raised here was lack of services, compounded by a lack of interest and expertise in discussing how to obtain help after discharge from hospital.

*“They will signpost you to the CMHT even when the CMHT refuse to see you”.*

*“…telling me to self-refer to IAPT service only to find out once I had done so they won’t take me on because I attempted suicide”.*

*“Suggested going to the local women’s centre, which I discovered had closed some time ago. Also suggested my local MIND but couldn’t make a referral or provide contact information”.*

Positive comments came from respondents who wrote about helpful contact with statutory services including crisis teams, addiction services and community mental health teams.

### Characteristics of a desirable service

Survey respondents were invited to detail the features of an ideal LMHS. Table 5 summarises the practical recommendations suggested by service users. Both service users and their partners, friends and families emphasised the importance of being treated with respect and compassion by a non-judgmental and supportive clinician who listens to them.

*“To treat everybody without judgment and with respect. To actually practise empathy”.*

**Table 5:** Recommendations from service users to improve acute care services and LMHS

	Recommendation
**1**	Patients should be seen more quickly in ED. Respondents felt that one hour should be the maximum somebody should wait if they have a significant mental health problem.
**2**	People should have an appropriate place to wait with food and drink available.
**3**	People should be assessed in a private room that is well-lit with comfortable chairs and of a reasonable size.
**4**	If people are agitated, they should receive monitoring whilst they are waiting. Staff should provide support and re-assurance during this period and encourage them to stay.
**5**	Staff should provide information for friends or family in the form of a written note, as the person may not be coherent enough to process verbal information at the time. The note should detail the person’s diagnosis, what treatment they have received and appropriate contact numbers and follow-up arrangements.
**6**	Staff should communicate clearly with patients and their families. Specifically, they should explain what to expect next and discuss options for immediate help and follow-up.
**7**	A psychiatrist, in addition to mental health nursing staff, should be available so that patients can receive a diagnosis and have medication prescribed if needed.
**8**	Patients with psychiatric symptoms secondary to a medical condition, such as steroid-induced psychosis, should not be transferred to a psychiatric ward but should be treated in the general medical setting or in a liaison mental health ward.
**9**	More psychological therapy should be available for patients in the general hospital with physical and mental health problems.
**10**	Respondents raised the possibility of respite care for people who are not severely ill enough to warrant admission to a psychiatric bed but not safe enough to go home alone.
**11**	Staff should be knowledgeable about the local services and third sector organisations available to patients and their families following discharge and should be helpful in facilitating contact.

Many respondents indicated that LMHS should review patients more quickly following referral from the medical team and spend more time face-to-face with the patient.

*“More time spent talking to patients to offer the support needed… Keeping in touch with nurses about waiting times”*.

A recurring complaint was that LMHS staff did not have up-to-date knowledge of the local services and third sector organisations available to patients following discharge. Respondents suggested that staff should be knowledgeable about these organisations and helpful in facilitating contact.

*“Suggested I should ring local Mind but didn’t have contact info and couldn’t explain what local Mind offered”.*

Clearer communication was also desired by many respondents. An improved service would provide information about what to expect next as well as the discussion of options for immediate help and follow-up.

*“Giving me more options than just ‘go into a psych ward’ or ‘go home’. I also felt that I had to choose to go home and that the option wasn’t really an option at all”*.

The partners, friends and families of service users highlighted that LMHS staff should take those accompanying the service user more seriously, as they know the patient best and feel more qualified to ascertain when they are off their baseline.

*“Listen to the people who knew him best”.*

Several also stressed the need for a 24-hour service for vulnerable people, detailing the additional difficulties faced by children and elderly patients.

*“Being able to access help anytime without it being an unbelievable battle for vulnerable people”.*

## Discussion

This is one of few evaluations of the experiences of LMHS by its users and close others in the UK. Both structured multiple choice and free-text answers presented a mixed picture with positive and negative experiences. Latent class analysis suggested that respondents either seemed to have a good experience of LMHS and felt supported and helped, or they had an unhelpful experience in which they were assessed or asked questions at a time when they felt unable to cope. Negative comments related mainly to organisational factors such as long waits and little after care, or to human factors such as uninterested or dismissive behaviour from staff.

Our survey was conducted fifteen years after the survey by Callaghan and colleagues [16], in the context of a drive from the government to assess, treat and discharge 95% of all patients seen in the emergency departments within four hours of their initial attendance. This approach to rapid assessment and discharge has, for example, been one of the main rationales for recent investment in LMHS. This national target would not have been in place at the time of the aforementioned studies. It is possible that this target, set up to address long waiting times, has had a detrimental effect on the quality of patient care delivered. Services are financially penalised for breaching these four-hour completion times and many have responded by introducing means of fast-tracking patients through the department, which may have led to some of the negative experiences described by our respondents.

The four-hour target is being replaced by a set of access standards, including the average waiting time in ED, time to initial clinical assessment, and time to emergency treatment for critically ill and injured patients [22]. At the time of writing, people in need or urgent mental healthcare will be seen within one hour of attending the ED by a member of the liaison team. Liaison mental health services in England are to be strengthened to meet this new target, with 70% of services meeting a core standard of staffing levels for size of hospital by 2023/24 and 100% coverage thereafter [22]. It remains to be seen if this new target for LMHS results in improved service user satisfaction.

As previously described in a recent systematic review, structural components of acute care can be experienced as cold or overstimulating [2], and these experiences were echoed by our respondents. In 2011 the Department of Health in England produced a framework of elements critical to patient’s NHS experience. These are: respect for patient-centred values, preferences and expressed needs; co-ordination and integration of care; information, communication and education; physical comfort; emotional support; welcoming and involving family and friends; and transition, continuity and access to care [23]. Our results suggest that many of these aspects of care are currently neglected for people with mental health problems in the acute setting. The Care Quality Commission (CQC), an independent body which inspects hospital services, rates more than half of all ED services as ‘inadequate’ or ‘requiring improvement’.

The reported long waits to be seen by a member of the LMHS are likely to be due to staffing levels. At the time of the survey only a minority of LMHS were staffed to a level allowing comprehensive 24/7 coverage [22]. Rude, dismissive or uninterested behaviour from staff is not something that can be explained by low staffing levels or work pressure and is clearly unacceptable. One reason for this finding may be the proportion of our respondents who had been seen after self-harm, which is known still, unfortunately to be associated with negative attitudes among staff [24]. Improvement in this area was, unsurprisingly, mentioned as an important area for improvement.

General attitudes towards mental health problems appear to be changing, although negative attitudes towards people who self-harm still exist. A public health campaign called ‘Time to Change’ designed to generate more positive attitudes towards mental health issues appears to have had a positive impact on the general population but less impact on the attitude of health professionals towards mental illness [25]. Mental health issues are talked about more openly in the media and by high profile figures. It is possible that people who attend ED in a mental health crisis have a greater expectation of care than in previous times and are consequently more dissatisfied when the care they receive is suboptimal. Work towards reducing negative attitudes towards individuals with mental health difficulties is important if we are to improve service users’ experiences of LMHS. Knaak and colleagues describe six active ingredients for programmes aimed at reducing stigma in healthcare settings, including contact-based education [26], although further research evaluating effective long-term strategies is warranted.

Our previous work on commissioning of liaison services has shown there is agreement across all NHS staff groups (commissioners, psychiatrists and hospital managers) that patients in mental health crises are currently being signposted to emergency departments, despite the concerns raised about the appropriateness of using EDs for this purpose [27]. A recent report by the Royal College of Emergency Medicine has recommended that all people presenting to EDs in a mental health crisis should undergo immediate mental health triage at the time of their arrival and that parallel physical and mental assessments should be the norm to improve patient experience and limit time in the emergency setting [28].

Given that LMHS frequently provide one-off contact with individuals during a time of mental health crisis before signposting or referring to the most appropriate service, good knowledge of local services and third sector organisations is essential for LMHS professionals. Our results indicate that many respondents were not signposted to such services, and a lack of up-to-date familiarity with services was identified as a theme in free-text responses. This is an area were staff training and supervision is needed to improve services.

Only one participant was aged over 65 years. We recommend that the importance of a separate LMHS for older adults is explored amongst a study sample of older adults who would be directly influenced by any changes to service provision for adults aged 65 and over.

This study has several strengths. This is one of the largest surveys of user experiences of LMHS and our participants included women and men of all ages and users of both ED and ward liaison services. We were also able to capture the experiences of both service users and their partners, friends and family members who accompanied them.

There are several limitations to the study. The Facebook survey cannot be regarded as representative of most users of LMHS. Survey interaction measures indicate that large numbers of individuals saw the survey and took a closer link by clicking, but only a small proportion completed the survey. Additionally, as we collected data from one social media platform, our results may be biased towards users of that site. It is possible that people who had particularly memorable experiences with LMHS, either positive or negative, were more likely to complete the Facebook survey. This could contribute to a more polarised interpretation of service user experiences, with an under-representation of average or acceptable experiences.

Our results should be regarded as providing an indication of the sorts of concerns people have about their experience of LMHS rather than as providing a representative survey of experience. For example, feedback on LMHS via the Royal College of Psychiatrists’ Psychiatric Liaison Accreditation Network (PLAN), an independent quality improvement service, regularly reports more positive user experiences of LMHS [29].

## Conclusions

Close attention must be given to evaluating and improving LMHS both from the organisational perspective (waiting times, clinical environment) and from the human side of the clinical interaction (staff attitudes and behaviour) as this experience can be inherently therapeutic while having implications on engagement with treatment and future psychiatric care.

## Supplementary Information

**Additional file 1:**

## Data Availability

Data from this study are available by request from E.G. subject to appropriate terms and conditions.

## Abbreviations

LMHS: Liaison Mental Health Services
ED: Emergency Department
CMHT: Community Mental Health Team
