# Supplementary material for: Experiences of people seen in an acute hospital setting by a liaison mental health service: responses from an online survey
Source: BMC Health Serv Res. 2021 Oct 5;21:1050. doi: 10.1186/s12913-021-06974-4 (PMC8493711; doi:10.1186/s12913-021-06974-4)
Supplement: Supplementary file 1 — Additional file 1: [file 12913_2021_6974_MOESM1_ESM.docx]

**Appendix 1.** Online survey questionnaire.

1. I am: [someone who has used a liaison psychiatry service/a friend of someone who has used a liaison psychiatry service/partner or family member of someone who has used a liaison psychiatry service]

2. Which of the options below best applies to you? I went to hospital with: [a mental health problem/a physical health problem/consequences of self-harm]

2.a. Did you go to the hospital because you were worried about your mental health?

2.b. Did someone from the liaison psychiatry team speak to you in the Emergency Department?

2.c. Did someone from the liaison psychiatry team speak to you on a Ward?

2.d. Did someone from the liaison psychiatry team speak to you in the pre-operative assessment clinic?

2.e. If you did not speak with someone from liaison psychiatry, would you have liked to?

2.f. Who did you speak to from the liaison psychiatry team (either in the Emergency Department or on a ward)? Please tick any that apply: [mental health nurse/psychiatrist/allied mental health professional (e.g. occupational or physio therapist)/psychologist or psychological therapist/other/not sure/not applicable]

2.f.i. If you selected Other, please specify:

2.f.ii. Did the person from liaison psychiatry introduce themselves? (For example did they say they were a psychiatrist or mental health nurse?)

2.g. If you developed mental health problems during your stay was this brought to the attention of nursing and medical staff so that they could provide you with the right care and support?

2.h. Did the person from the mental health team explain why they had been called to see you?

2.i. At any point did you feel that you had to see someone from the mental health team before the other staff would arrange your discharge from hospital?

3. Was your contact with the liaison psychiatry service helpful?

4. Please tell us what, if anything, about your contact with the liaison psychiatry service that was useful to you?

4.a. What could have been done better, if anything, about your contact with liaison psychiatry?

4.b. What was good, if anything, about your contact with the liaison psychiatry service?

5. Did you have what is sometimes called a psychiatric assessment (where you are asked questions about your mental health and your feelings)?

5.a. How long after you arrived at the hospital did the psychiatric assessment happen? (Please indicate if this is hours or days)

5.b. Roughly how long did the assessment take?

5.c. Did you think there was a possibility that that you would not be able to get treatment for your physical health problem unless you saw someone from the liaison psychiatry team first?

5.d. Was the psychiatric assessment done in a private room/space?

5.e. Were the questions in the assessment asked at a time when you were able to answer them properly?

5.f. Were you able to answer most of the questions in the psychiatric assessment?

5.g. Did you feel that the member of staff who did the psychiatric assessment understood you and what you needed?

5.h. Did the person who did the psychiatric assessment make you feel comfortable enough for you to be honest with them about how you were feeling?

6. Was your assessment by the liaison psychiatry service helpful?

7. Please tell us what, if anything, about your assessment by the liaison psychiatry service that was useful to you?

7.a. What could have been done better, if anything, about your assessment by the liaison psychiatry service?

7.b. What was good, if anything, about your assessment by the liaison psychiatry service?

8. If you were moved to a ward or another department did you understand what was happening?

9. Did you get offered support or advocacy from outside of your wider medical team? (In other words, from someone who could help you understand your choices without offering an opinion or judgement?)

9.a. If support or advocacy was offered and you would like to tell us more about it, or if it was not offered and you would like to comment on this, please do so in the box below

10. Was an appointment as an outpatient offered after you left the hospital?

10.a. If you were offered an appointment as an outpatient after you left the hospital please indicate the type of clinic this was for: [medical outpatients/surgical outpatients/community mental health/liaison psychiatry outpatients/plastic surgery/pain clinic/fracture clinic/other]

10.a.i. If you would like to tell us more about your outpatient experience, please do so in the box below.

10.a.ii. If you selected Other, please specify:

11. Was your mental health recorded on your discharge paperwork (for example a letter to your GP) as part of planned care approach?

12. Were you given information about help that you could get from other services (for example addiction services, support groups, etc.)?

12.a. If you would like to tell us more about support you were offered from other services, please do so in the box below.

12.b. If you were given any information about other services or support available, was it...? [helpful/unhelpful/not sure/not applicable]

12.c. Please tell us which, if any, of the outpatients or aftercare was useful to you?

12.c.i. What could have been done better, if anything, about the outpatients or aftercare?

12.c.ii. What was good, if anything, about the outpatients or aftercare?

13. The list below contains features which some but not all liaison psychiatry services have. Which one feature do you think is or would be the most important to you? [offering outpatient follow-up by the liaison psychiatry team if needed after you leave hospital/liaison psychiatry sharing more information with doctors and nurses caring for your physical health/providing a 24 hour 7 day service/having a mix of psychiatrists, mental health nurses and other professionals such as counsellors, psychologists and occupational therapists with different skills in the liaison psychiatry team/making sure that all liaison psychiatry services are the same no matter where you are in England/having target response times (for example seeing patients within 4 hours of a referral)/having a separate liaison psychiatry service for older people]

14. The list below contains features which some but not all liaison psychiatry services have. Which one feature do you think is or would be the least important to you? [offering outpatient follow-up by the liaison psychiatry team if needed after you leave hospital/liaison psychiatry sharing more information with doctors and nurses caring for your physical health/providing a 24 hour 7 day service/having a mix of psychiatrists, mental health nurses and other professionals such as counsellors, psychologists and occupational therapists with different skills in the liaison psychiatry team/making sure that all liaison psychiatry services are the same no matter where you are in England/having target response times (for example seeing patients within 4 hours of a referral)/having a separate liaison psychiatry service for older people]

15. Please use the box below to tell us more about what you think the priorities of liaison psychiatry services should be

16. The last time I went to a general hospital and spoke to someone from liaison psychiatry was (estimated)

17. My age is: [18-24 years/25-34 years/45-54 years/55-64 years/65+ years]

18. The gender I identify with is: [male/female/would rather not say]
